# Analytical variables influencing the performance of a miRNA based laboratory assay for prediction of relapse in stage I non-small cell lung cancer (NSCLC)

**DOI:** 10.1186/1756-0500-4-424

**Published:** 2011-10-19

**Authors:** Jesper Dahlgaard, Wiktor Mazin, Thomas Jensen, Mette Pøhl, Wiam Bshara, Anker Hansen, Eric Kanisto, Stephen Jacques Hamilton-Dutoit, Olfred Hansen, Henrik Hager, Henrik J Ditzel, Sai Yendamuri, Steen Knudsen

**Affiliations:** 1 Medical Prognosis Institute, Venlighedsvej 1, 2970 Hørsholm, Denmark; 2 Department of Thoracic Surgery, Roswell Park Cancer Institute, Buffalo, New York, USA; 3 Department of Oncology, Odense University Hospital, Sdr. Boulevard 29, DK-5000 Odense C, Denmark; 4 Department of Cancer and Inflammation Research, Institute for Molecular Medicine (IMM), University of Southern Denmark, J. B. Winsloews Vej 25, DK-5000 Odense C, Denmark; 5 Institute of Pathology, Aarhus University Hospital, Noerrebrogade 44, Bygning 18, DK-8000 Aarhus C, Denmark; 6 Department of Pathology, Roswell Park Cancer Institute, Buffalo, New York, USA

## Abstract

**Background:**

Laboratory assays are needed for early stage non-small lung cancer (NSCLC) that can link molecular and clinical heterogeneity to predict relapse after surgical resection. We technically validated two miRNA assays for prediction of relapse in NSCLC. Total RNA from seventy-five formalin-fixed and paraffin-embedded (FFPE) specimens was extracted, labeled and hybridized to Affymetrix miRNA arrays using different RNA input amounts, ATP-mix dilutions, array lots and RNA extraction- and labeling methods in a total of 166 hybridizations. Two combinations of RNA extraction- and labeling methods (assays I and II) were applied to a cohort of 68 early stage NSCLC patients.

**Results:**

RNA input amount and RNA extraction- and labeling methods affected signal intensity and the number of detected probes and probe sets, and caused large variation, whereas different ATP-mix dilutions and array lots did not. Leave-one-out accuracies for prediction of relapse were 63% and 73% for the two assays. Prognosticator calls ("no recurrence" or "recurrence") were consistent, independent on RNA amount, ATP-mix dilution, array lots and RNA extraction method. The calls were not robust to changes in labeling method.

**Conclusions:**

In this study, we demonstrate that some analytical conditions such as RNA extraction- and labeling methods are important for the variation in assay performance whereas others are not. Thus, careful optimization that address all analytical steps and variables can improve the accuracy of prediction and facilitate the introduction of microRNA arrays in the clinic for prediction of relapse in stage I non-small cell lung cancer (NSCLC).

## Background

Early stage non-small cell lung (NSCLC) cancer is characterized by both clinical and molecular genetic heterogeneity with five-year recurrence and survival rates of 50% and 73% respectively [1]. Although several randomized studies have been performed, the use of adjuvant chemotherapy for stage I NSCLC still is controversial [2] and surgical resection remains the primary treatment for this disease.

However, in spite of tumor heterogeneity, new techniques in molecular profiling [3-5] can supplement clinical and pathologic observations and help to identify patients with a particularly poor prognosis. This can be useful both for intensified follow-up and for administering therapy specifically to patients at a high risk of recurrence [6].

In this study, we performed global microarray expression profiling targeting several small non-coding RNA species including microRNAs (miRNAs). MicroRNAs are small noncoding RNAs of approximately 18-25 nucleotides in length that regulate gene expression at the post transcriptional level by base pairing with mRNA, leading to either translational repression [7], or mRNA degradation [8-10]. MicroRNAs have been estimated to regulate up to 30% of all human genes [11], and frequently reside in cancer associated genomic regions [12]. Deregulation of miRNA expression plays a direct role in oncogenesis, and in differentiation and progression in cancer, in part because deregulation can change the expression of oncogenes and tumour suppressor genes [13]. Strong deregulation of miRNA expression has been seen in several forms of cancer, including lung carcinoma [4], and several studies have suggested that miRNA profiling can be used for prognostication in lung cancer [3-6].

The enhanced stability of microRNAs in contrast to mRNAs, allow expression profiling in routinely stored formalin-fixed and paraffin-embedded (FFPE) specimens, including samples that are more than ten years old [14]. Large FFPE archives exist in diagnostic pathology departments throughout the world. When linked to clinical data, they represent an invaluable biobank resource for exploring the association between molecular changes in tumors and clinical endpoints such as relapse or survival after surgery. Furthermore in the case of early stage NSCLC, FFPE specimens will be available for most patients. Therefore, it is realistic to use miRNAs and non-coding RNAs as biomarkers for prognosis in stage I NSCLC, once a prognostic signature has been clinically validated.

In order to reach this goal, carefully conducted studies are needed [15,16], incorporating well defined experimental procedures that may eventually lead to the development of clinically validated applications allowing for individual treatment strategies in early stage NSCLC. Previously, the Microarray Quality Control (MAQC) study [17] focused on the entire process from sample handling, through laboratory and assay conditions, to data normalization and bioinformatics. This demonstrated the scope and significant potential of microarray technology for the clinic [18] when performed under careful and well-defined experimental conditions.

**Figure 1:**
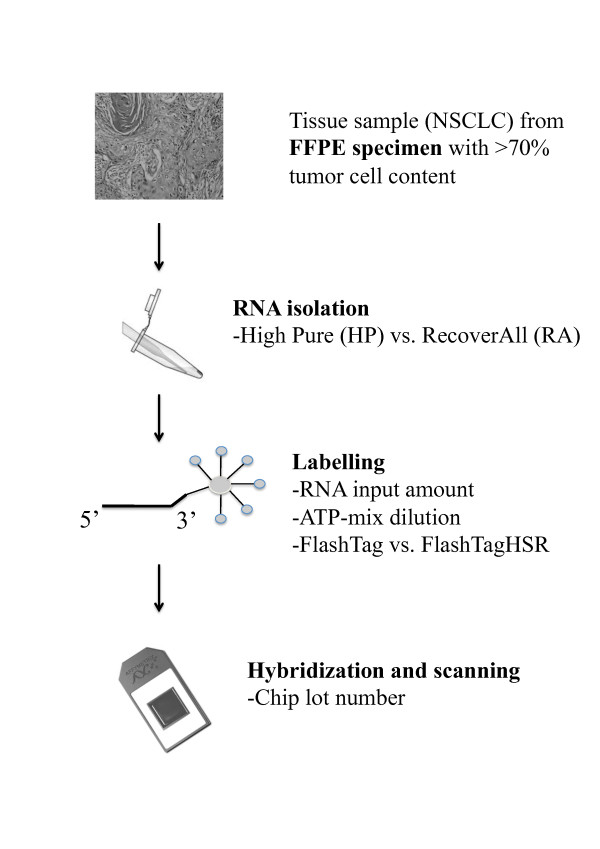
**Work-flow starting by securing the tumor tissue from the FFPE specimen and ending by scanning the array, including the different analytical variables under study**: i) *RNA extraction kit*, ii) *RNA input amount*, iii) *ATP-mix dilution*, iv) *RNA labeling kit*, and v) *chip lot number*.

In this study, we compared two laboratory assays for prognostication in stage I NSCLC based on miRNA profiling in FFPE tissue specimens. To perform an objective evaluation [16], of the different reagents, array products and protocols we examined several analytical conditions (figure 1) including: i) 7 different *RNA input amounts *using one RNA preparation of a single tumor specimen, ii) three different *ATP-mix dilutions *using two RNA preparations of two tumor specimens, iii) two different *array lot numbers *using one RNA preparation of a single tumor specimen, iv) two different *RNA extraction kits *using eight RNA preparations of four tumor specimens, and, v) two different *RNA labeling kits *using four RNA preparations of four tumor specimens in 8 labeling reactions. In addition, RNA was extracted twice from the same specimens in a cohort of more than 60 NSCLC patients in a direct comparison of the two assays. Thus, 139 RNA extractions and 166 hybridizations were performed from a total of 75 NSCLC specimens. To qualify the impact of any variation in the assay specific analytical conditions, principal component analysis was performed. In addition, prognosticator calls (i.e. "recurrence" or "no recurrence") was examined after varying the analytical conditions for selected samples.

## Results

### RNA input amount

**Figure 2:**
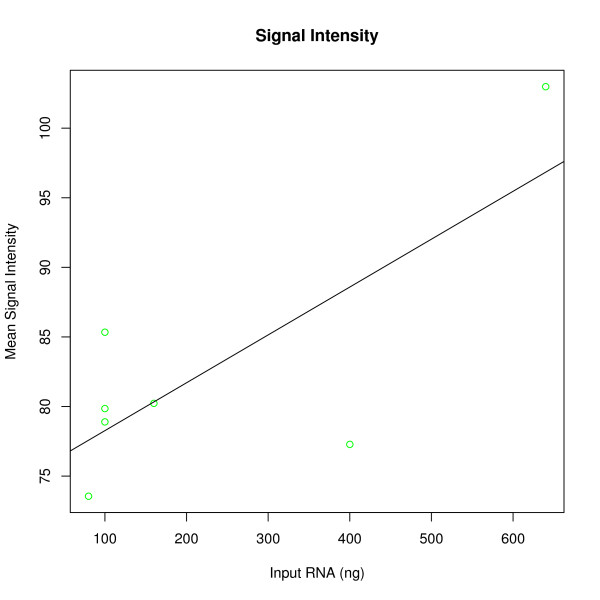
**Mean signal intensity using varying input amounts (ng) of small RNA from an NSCLC T2 tumor that was preserved in RNA*later *within 2 hours of surgery**.

**Figure 3:**
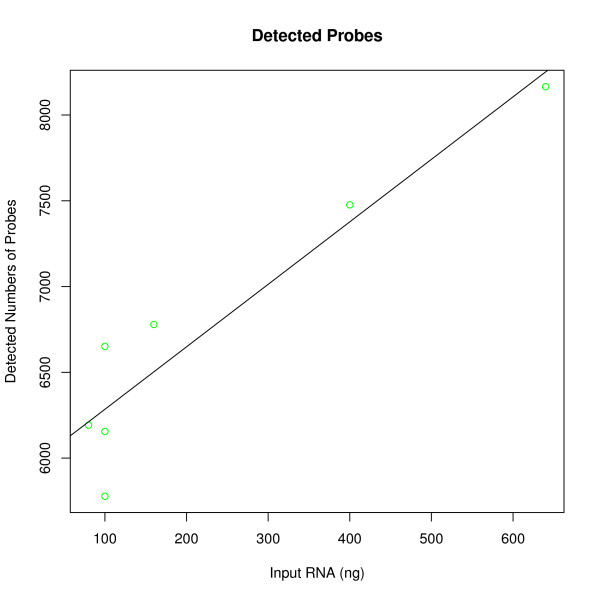
**Number of detected probes using varying input amounts (ng) of small RNA from an NSCLC T2 tumor that was preserved in RNA*later *within 2 hours of surgery**.

**Figure 4:**
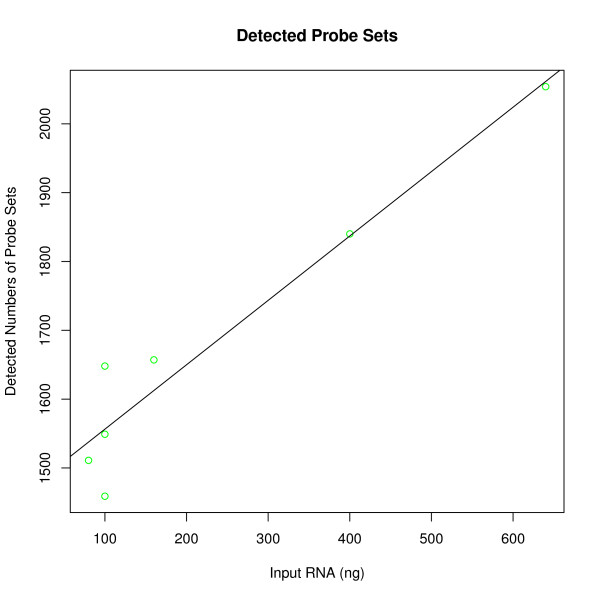
**Number of detected probe sets using varying input amounts (ng) of small RNA from an NSCLC T2 tumor that was preserved in RNA*later *within 2 hours of surgery**.

**Figure 5:**
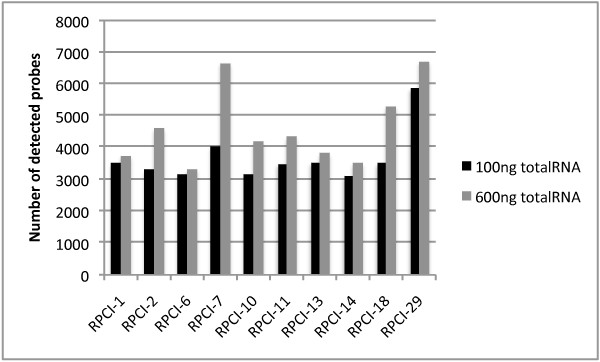
**Number of detected probes in hybridizations where tumor samples were labeled using either 100 ng or 600 ng total RNA**.

**Figure 6:**
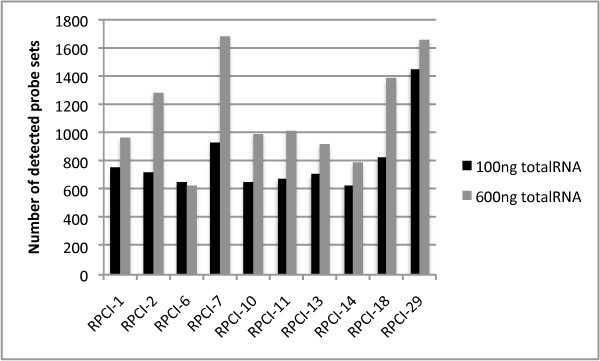
**Number of detected probe sets in hybridizations where tumor samples were labeled using either 100 ng or 600 ng total RNA**.

**Figure 7:**
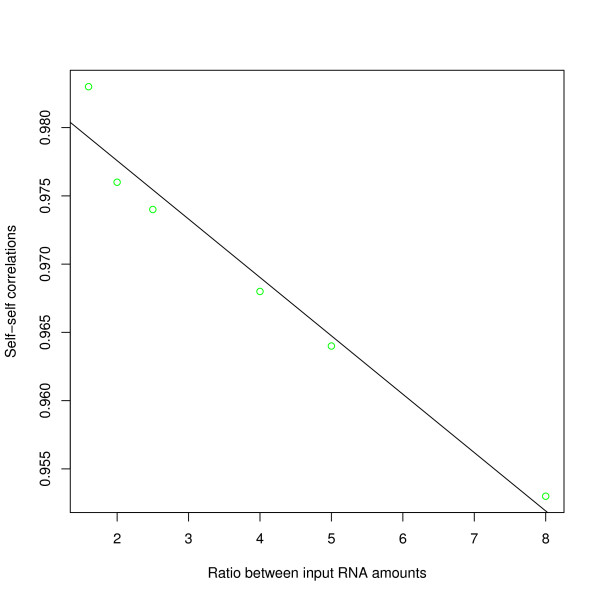
**Linear regression analysis revealed that self-self correlations in probe signal intensities between arrays hybridized to different amounts of RNA from an NSCLC T2 tumor (preserved in RNA*later*) decreased when deviations in RNA amounts between pairs increased, considering all pair wise combinations (*b*_*DevRNAinput*_ = 0.0002 ± 0.00002, t = -11.7, p < 0.001, R^2^ = 0.96)**.

A linear regression model showed that the amount of purified small RNA used for hybridization significantly affected mean signal intensity; the number of detected probes; and the number of detected probe sets (*b*_*signal*_ = 0.03 ± 0.01, t = 2.6, p < 0.05, R^2^ = 0.58; *b*_*probes*_ = 3.64 ± 0.60, t = 6.0, p < 0.01, R^2^ = 0.88; *b*_*probe sets*_ = 0.94 ± 0.12, t = 7.6, p < 0.001, R^2^ = 0.88; figure 2, 3 and 4). The amount of total RNA used for hybridization also affected the number of detected probes (mean_100 ng_ = 3674 vs. mean_600 ng_ = 4627, t = -2.04, df = 18, p = 0.05; figure 5) and the number of detected probe sets (mean_100 ng_ = 803 vs. mean_600 ng_ = 1134, t = -2.41, df = 18, p < 0.05; figure 6). Self-self correlations in probe signal intensities between arrays hybridized to different amounts of the same RNA preparation varied across different combinations of RNA input amount (table 1). In addition, a linear regression analysis revealed that self-self correlations in probe signal intensities decreased when ratios in RNA input amounts between pairs increased, considering all pair wise combinations (*b*_*DevRNAinput*_ = -0.0042 ± 0.0004, t = -10.0, p < 0.001, R^2^ = 0.95; figure 7).

**Table 1:** Correlations in signal intensities across probes from different arrays that were hybridized to four different amounts of RNA (ATP-mix dilution, 1:50) from a single preparation of a T2 NSCLC tumor.

RNA amount	80 ng	160 ng	400 ng	640 ng
80 ng	-	0.976	0.964	0.953
160 ng	-	-	0.974	0.968
400 ng	-	-	-	0.983
640 ng	-	-	-	-

### ATP-mix dilution

The effect of ATP-mix dilution was not significant in a linear regression model (results not shown), when analyzing six hybridizations with RNA from two NSCLC specimens, each labeled using three different ATP-mix dilutions. Thus, mean signal intensity, background intensity, the numbers of detected probes, and the numbers of detected probe sets were stable across the tested range (table 2). Self-self correlations coefficients in probe signal intensities between arrays with RNA labeled at different ATP-mix dilutions were invariant across the tested range (table 3). Thus, there were no association between self-self correlations and the ratio of ATP-mix dilutions among arrays hybridized to RNA labeled at different ATP-mix dilutions, considering all pair wise combinations (results not shown).

**Table 2:** Mean( ± SEM) signal intensity, background intensity, number of detected probes and number of detected probe sets in hybridizations using 600 ng RNA from two NSCLC specimens (duplicates) each labeled using three different ATP-mix dilutions (1:50; 1:150 and 1:500).

ATP-mix dilution	1:50	1:150	1:500
Mean Intensity	349 ± 27	339 ± 19	351 ± 9
Mean Background Intensity	89.3 ± 0.5	97.6 ± 4.6	92.3 ± 3.7
Number of Detected Probes	9762 ± 213	9378 ± 435	9585 ± 139
Number of Detected Probe Sets	2432 ± 28	2382 ± 101	2371 ± 12

**Table 3:** Self-self correlations (± SD) in signal intensities across probes in six hybridizations using 600 ng RNA from two NSCLC specimens with each specimen labeled with three different ATP-mix dilutions (1:50; 1:150 and 1:500).

ATP-mix dilution	1:50	1:150	1:500
1:50	-	0.990 ± 0.001	0.990 ± 0.0
1:150	-	-	0.989 ± 0.001
1:500	-	-	-

### Different chip lot numbers

Hybridizations (in triplicates) with labeled RNA from a single T2 NSCLC tumor revealed that signal intensity and the number of probes and probe sets were not significantly different across different lot numbers (results not shown). In addition, the observed self-self correlation coefficient across probe signal intensities *within *and *between *lots did not vary (table 4). In particular, the self-self correlation coefficient across probe signal intensities *within *one lot of arrays (cc = 0.973) was similar to the estimated average correlation *between *two different lots of arrays (cc = 0.965; 95% C.I. = 0.92-1.01).

**Table 4:** Correlations in signal intensities across probes from two different lots of arrays that were hybridized (in triplicates) to 100 ng of labeled RNA (ATP-mix dilution 1:50) from of a single RNA preparation of a T2 NSCLC tumor.

Array	Chip 1, Lot "A"	Chip 2, Lot "A"	Chip 3, Lot "B"
Chip 1, Lot "A"	-	0.973	0.968
Chip 2, Lot "A"	-	-	0.961
Chip 3, Lot "B"	-	-	-

### Comparisons of two different RNA extraction kits

**Figure 8:**
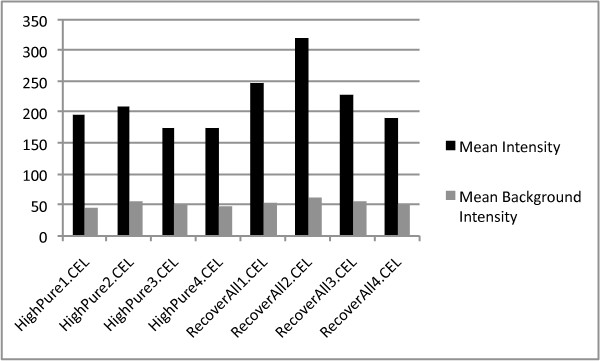
**Mean signal and background intensities in hybridizations using RNA prepared from four tumor samples using both the HighPure and RecoverAll RNA extraction kits for FFPE studies**.

**Figure 9:**
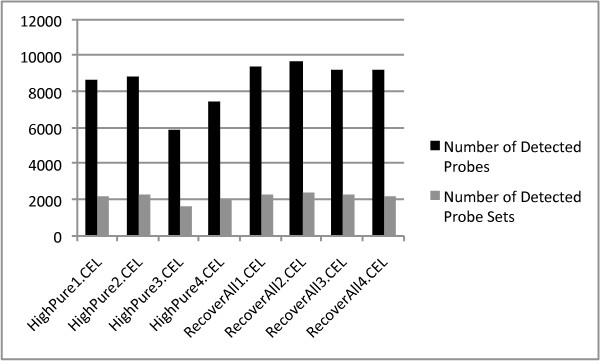
**Number of detected probes and probe sets in hybridizations using RNA prepared from four tumor samples using both the HighPure and RecoverAll RNA extraction kits for FFPE studies**.

**Figure 10:**
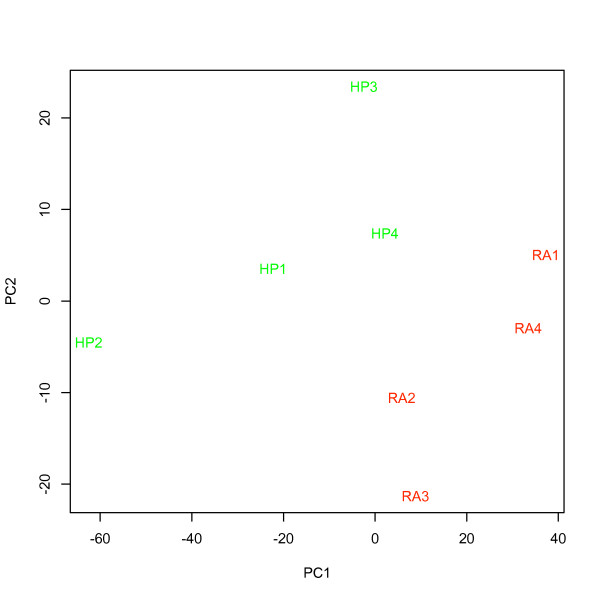
**PCA for all human non-coding RNAs in hybridizations using RNA prepared from four tumor samples using both the HighPure (Roche) and RecoverAll (Ambion) RNA extraction kits for FFPE studies**. "HP" (green) represents samples extracted using HighPure (Roche), and, "RA" (red) represents the same samples extracted using RecoverAll (Ambion).

**Figure 11:**
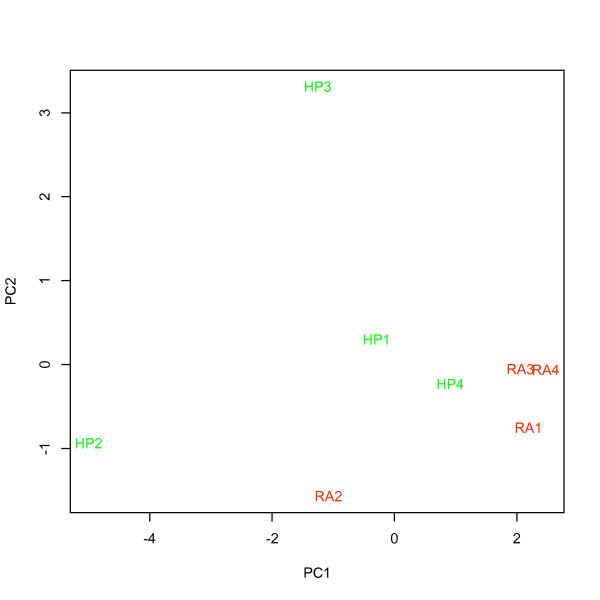
**PCA for a 12 gene signature for prognosis in NSCLC in hybridizations using RNA prepared from four tumor samples using both the HighPure (Roche) and RecoverAll (Ambion) RNA extraction kits for FFPE studies**. "HP" (green) represents samples extracted using HighPure (Roche), and, "RA" (red) represents the same samples extracted using RecoverAll (Ambion).

Mean intensity ± se (x_RecoverAll_ = 247.7 ± 26.9 vs. x_HighPure_ = 190.1 ± 8.2), the number of detected probes ± se (x_RecoverAll_ = 9407.8 ± 98.0 vs. x_HighPure_ = 7733.5 ± 671.1) and the number of detected probe sets ± se (x_RecoverAll_ = 2328.8 ± 27.9 vs. x_HighPure_ = 2088.5 ± 150.0) in hybridizations with total RNA extracted using the *RA kit *all exceeded that for the *HP kit *(figure 8 and 9), although this was significant only for the numbers of detected probes (ANOVA; F_1,6_ = 6.09, P < 0.05). Background intensity was not significantly different between the kits (x_RecoverAll_ = 56.3 ± 2.5 vs. x_HighPure_ = 51.7 ± 2.0; figure 8). PCA, considering the expression of all human ncRNAs, as well as that of a specific miRNA signature for prognostication, demonstrated that a major proportion of the variance could be assigned to the two RNA extraction methods (i.e. *between-kit *variance) as revealed by the first principal component (PC1; figure 10 and 11).

### Comparisons of two different RNA labeling kits

**Figure 12:**
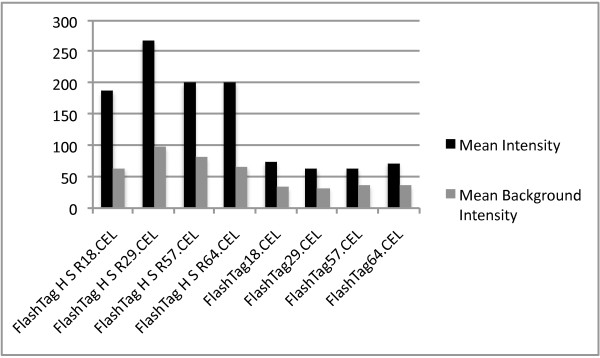
**Mean signal and background intensities in hybridizations using RNA from four tumor samples labeled with the FlashTag Biotin- and FlashTag Biotin HSR labeling kits**.

**Figure 13:**
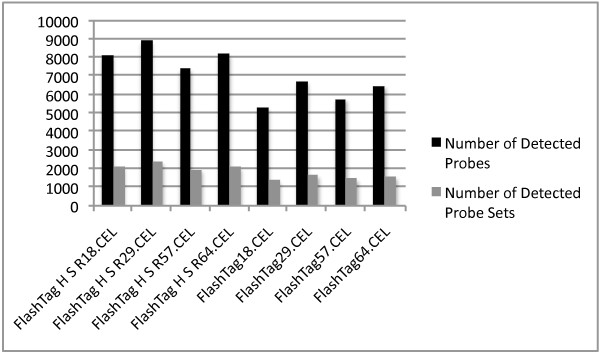
**Number of detected probes and probe sets in hybridizations using RNA from four tumor samples labeled with the FlashTag Biotin- and FlashTag Biotin HSR labeling kits**.

**Figure 14:**
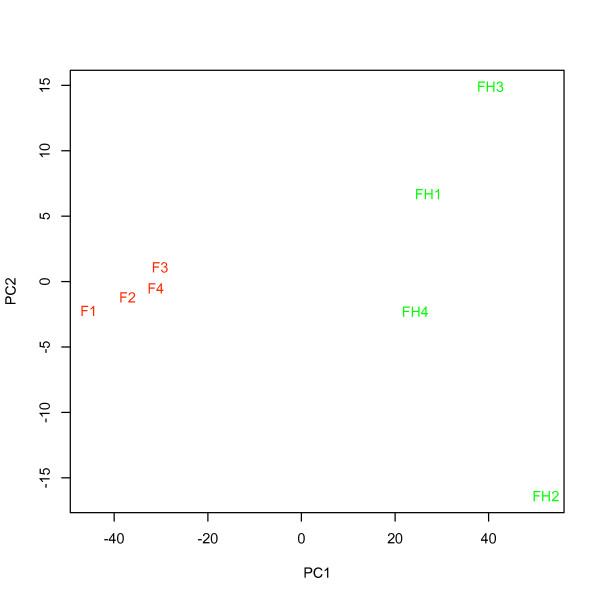
**PCA for all human non-coding RNAs in hybridizations using RNA from four tumor samples labeled with both the FlashTag Biotin- and FlashTag Biotin HSR labeling kits**. F (red) represents samples labeled with the FlashTag Biotin kit, and, FH (green) represents the same samples labeled with the FlashTag Biotin HSR labeling kit.

**Figure 15:**
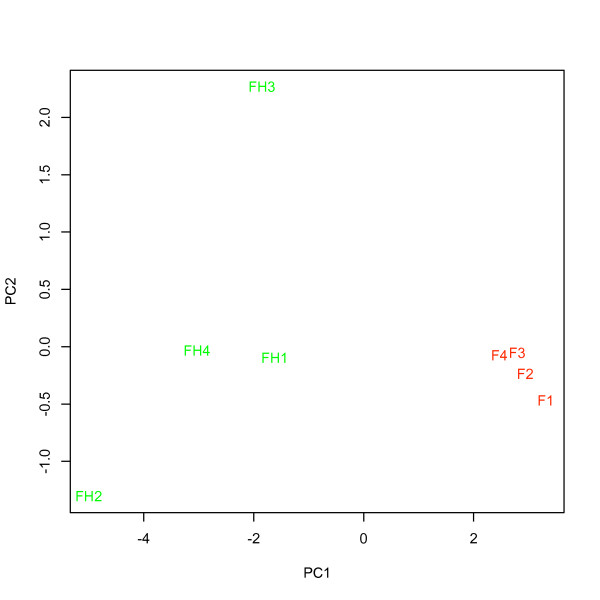
**PCA for a 12 gene signature for prognosis in NSCLC in hybridizations using RNA from four tumor samples labeled with both the FlashTag Biotin- and FlashTag Biotin HSR labeling kits**. F (red) represents samples labeled with the FlashTag Biotin kit, and, FH (green) represents the same samples labeled with the FlashTag Biotin HSR labeling kit.

For hybridizations with RNA labeled using the *FlashTag Biotin HSR labeling kit*; mean intensity (x ± se _*FlashTag HSR*_ = 215.1 ± 18.1 vs. x ± se _*FlashTag*_ = 68.3 ± 2.9; ANOVA; F_1,6_ = 64.4, P < 0.001), the numbers of detected probes (x ± se _*FlashTag HSR*_ = 8153 ± 302 vs. x ± se *_FlashTag_* = 6079 ± 323; ANOVA; F_1,6_ = 21.9, P < 0.01) and the numbers of detected probe sets (x ± se _*FlashTag HSR*_ = 2139 ± 87 vs. x ± se *_FlashTag_* = 1527 ± 61; ANOVA; F_1,6_ = 33.5, P < 0.01), all exceeded those in hybridizations with RNA labeled using the *FlashTag Biotin labeling kit *(figure 12 and 13). Background intensity was significantly different between the kits (x ± se _*FlashTag HSR*_ = 77.6 ± 8.0 vs. x ± se *_FlashTag_* = 34.8 ± 0.9; ANOVA; F_1,6_ = 28.1, P < 0.01). A major proportion of the variance could be assigned to the different labeling methods (*between-kit *variance) as revealed by PC1 in the PCA (figure 14 and 15). PCA also revealed that the variance for samples labeled with the old labeling methods was very small (compressed).

### Assay I and II for prognostication in stage I NSCLC samples

**Figure 16:**
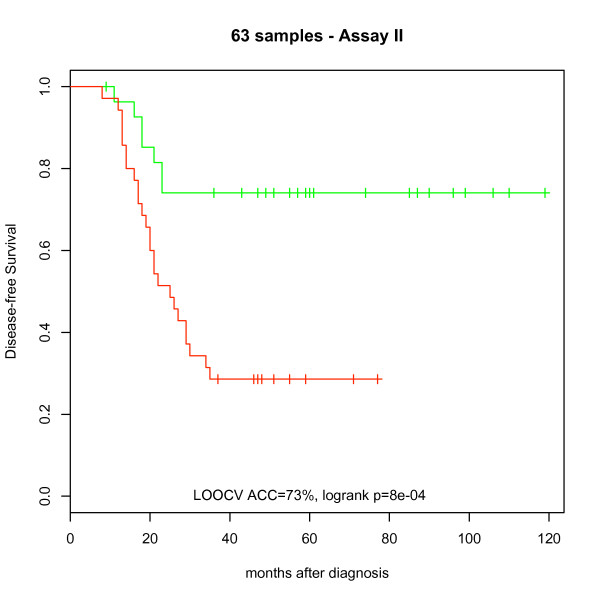
**Kaplan-Meier plots of disease-free survival predictions (n = 63) in a leave-one-out cross-validation analysis (using the SVM-based classifier)**. Cases predicted to have recurrence are plotted separately (red) from those predicted to be recurrence-free (green).

By performing 1000 Monte Carlo simulations we obtained a prognostic accuracy of 60.0% (95% C.I.: 59.5% - 60.5%) for assay I and 62.6% (95% C.I.: 61.9% - 63.2%) for assay II (p = 9.82e-10 for the hypothesis that the accuracy is similar for the two assays). Nested LOOCV that optimized the number of selected non-coding RNAs in a separate loop resulted in an LOOCV accuracy of 63% for assay I and 73% for assay II. A multivariate analysis examined for the effects of the miRNA chip based prognosis (i.e. "recurrence" or "no recurrence"), age, smoking status, stage (Ia or Ib) and histology (squamous or adeno) on recurrence after surgery. Only the miRNA based prognosticator was significant (P_miRNA Prognosis_ = 0.009; P_Age_ = 0.656, P_Smoking_ = 0.146, P_Stage_ = 0.921, P_Histology_ = 0.732). Figure 16 shows the predictions against a Kaplan-Meier time-to recurrence plot (LOOCV accuracy of 73%, p < 0.001). The two miRNA lists obtained did not overlap and the list obtained from one assay could not predict the other assay.

### Impact of the analytical conditions on the robustness of the prognosticator

Prognosticator calls (i.e. "recurrence" or "no recurrence") were consistent independent of the RNA amount, ATP-mix dilution, chip lot number and RNA extraction method being used. In contrast, the calls were not robust to changes in labeling method (table 5).

**Table 5:** Prognosticator calls (0 ="no recurrence" or 1 = "recurrence") were examined for varying RNA input amounts (a single T2 NSCLC specimen), ATP-mix dilutions (two NSCLC specimens*)*, chip lot numbers (a single T2 NSCLC specimen), RNA extraction kits (four NSCLC specimens*; HP = HighPure*, Roche &*RA = RecoverAll*, Ambion) and RNA labeling kits (four NSCLC specimens*; F = Flashtaq *and *FH = Flashtaq HSR*, Genisphere) for selected samples using the prognostic profile of assay II.

*RNA amount*	*Call*	*Recurrence*	***Chip lot***	*Call*	*Recurrence*	*Labeling kit*	*Call*	*Recurrence*
80 ng	1	-	Lot *x*	1	-	RPCI-18 *- F1*	1	No
100 ng	1	-	Lot *x*	1	-	RPCI-29 *- F2*	1	Yes
100 ng	1	-	Lot *y*	1	-	RPCI-57 *- F*3	1	No
100 ng	1	-				RPCI-64 *- F*4	1	No
160 ng	1	-						
400 ng	1	-	***RNA extraction kit***			RPCI-18 *- FH1*	0	No
640 ng	1	-				RPCI-29 *- FH2*	0	Yes
			KH882A9 - *HP*	0	-	RPCI-57 *- FH3*	0	No
***ATP-mix dilution***			KH22716D3 - *HP*	0	-	RPCI-64 *- FH4*	0	No
			KH24218B4 - *HP*	0	-			
A-504; 1:50	0	-	KH24935B5 - *HP*	0	-			
A-504; 1:150	0	-						
A-504; 1:500	0	-	KH882A9 - *RA*	0	-			
B-652; 1:50	0	No	KH22716D3 - *RA*	0	-			
B-652; 1:150	0	No	KH24218B4 - *RA*	0	-			
B-652; 1:500	0	No	KH24935B5 - *RA*	0	-			

Data for recurrence was available only for the test of labeling kit and for one sample used to test ATP-mix dilution.

## Discussion

Validation of a microarray based laboratory assay poses two technical challenges; first, ensuring that data are aquired with the best laboratory proficiency; and second, that data are analyzed appropriately. In order for a chip based prognostic assay to be practically usefull and accurate for prognostication in NSCLC, concern must be adressed towards the concordance of expression measurements and the impact of variation across analytical conditions. Here we assessed the impact of variation in several analytical conditions including varying RNA input amount, ATP-mix dilution, chip lot numbers, RNA extraction- and RNA labeling kit.

### RNA input amount

Increasing the input RNA amounts led to an increase in mean signal intensity and the number of detected probes and probe sets (figure 2, 3, 4 and 5). Since no amplification step is applied in either of the labeling kits under test, this finding is expected. Also, as deviations in RNA input amounts affected self-self correlations, concern should be addressed to avoid large variations in the amount of input RNA in similar miRNA based laboratory assays (figure 7).

### ATP-mix dilution

When 600 ng RNA (obtained with the *RA kit*) was used as input in labeling reactions, ATP-mix dilutions did not significantly affect mean signal intensity and the number of detected probes and probe sets. Self-self correlations in probe signal intensities between arrays were also not affected by changing ATP-mix dilutions (table 3). Thus experimental variation in ATP-mix dilutions appear to have no impact.

### Different chip lot numbers

Correlations in signal intensities (table 4) were not affected by different lot numbers of arrays. This result was anticipated in part due to the In Vitro Diagnostics status of the Affymetrix gene array scanner being used here. In addition, Wen et *al. *[19] have demonstrated that even for arrays that were expired by several years (and of different lot numbers) the percentage of overlap between lists of differentially expressed genes from the expired and unexpired microarrays was 96.99%. In addition, microarray data generated using the expired microarrays were highly concordant with microarray and TaqMan^®^ data generated by the MAQC project several years before [19].

### Comparison of the HighPure and RecoverAll purification kits for FFPE studies

Mean intensity and the number of detected probes and probe sets, in RNA preparations from the *RA *extraction kit, all exceed that detected in RNA preparations obtained from the *HP *extraction kit. This is consistent with previous findings [14], showing that miRNA expression signals may be reduced when RNA is extracted using the *HighPure *miRNA isolation kit (Roche). Doleshal et *al*. [14], compared five different miRNA extraction kits and concluded that three of the kits showed two- to three-fold lower total RNA yield, and five- to 20-fold lower miRNA qRT-PCR signals at equal RNA mass input compared to alternative extraction kits, including *RecoverAll *(Ambion). PCA for all human non-coding RNAs revealed substantial variation *between *the two extraction kits. Thus, the results of the PCA indicate that a particular tumor sample is more distantly separated from itself when RNA is extracted with a different kit, as compared with tumor samples from different patients when RNA is extracted with the same kit. As a consequence, the two RNA extraction procedures are not interchangeable within either an experiment, or across different experiments that are performed for validation purposes.

### Comparison of the FlashTag Biotin and FlashTag Biotin HSR labeling kits

Mean intensity and the number of detected probes and probe sets, in RNA preparations labeled with the *FlashTag Biotin HSR labeling kit*, all exceed those in RNA preparations labeled with the original *FlashTag Biotin labeling kit*. PCA for all human non-coding RNAs revealed substantial variation *between *the two labeling kits. The results of the PCA indicate that a particular tumor sample is more distantly separated from itself when RNA is labeled with a different kit, as compared to tumor RNA samples from different patients labeled with the same kit. *Within-kit *variance for the *FlashTag Biotin labeling kit *was very small compared to the *FlashTag Biotin HSR labeling kit. *The small variance for the *FlashTag Biotin labeling kit *may be a consequence of a compressed mean (and variance) in signal intensity (figure 12), in addition to a reduced detection of probes and probes sets with this kit compared to the *FlashTag Biotin HSR labeling kit *(figure 13), when the same samples were processed. As a consequence, the two RNA labeling procedures are not interchangeable either within an experiment, or across different experiments that are performed for validation purposes.

### Comparisons of Assays I & II

By comparing assay I and assay II using a profile of fixed size (i.e. 30 non-coding RNAs) for each assay, and performing 1000 Monte Carlo simulations, a significantly better performance of assay II (62.6%) was observed as compared to assay I (60%). The accuracy of the final profiles (after performing nested LOOCV) for assay I and assay II were 63% and 73% respectively, again pointing to a better performance of assay II. A Kaplan-Meier time-to recurrence plot using data from assay II demonstrated a clear and significant separation of the predicted "recurrence" and "no recurrence" groups (LOOCV accuracy of 73%, p < 0.001; figure 16). The miRNA list obtained on one assay, however, could not predict NSCLC samples profiled using the other assay adding to the importance of extraction and labeling kits on performance of miRNA based classifiers. The observed accuracy of assay II was lower compared to an assay developed using the Exiqon platform that demonstrated an accuracy of 83%, in spite of the same patient samples being used in both studies [6]. This may in part be due to the larger number of samples being assayed in the study using the Exiqon platform [6]. Even though the profiles of the two platforms are not identical, the assay in Patnaik et *al*. [6] and assay II maintained a high accuracy, which is consistent with the MAQC studies that demonstrated that data quality from single- and two color platforms was essentially equivalent [20].

### Impact of the analytical conditions on the robustness of the prognosticator

The prognosticator calls (i.e. "recurrence" or "no recurrence") for selected samples under varying analytical conditions (table 5) were consistently independent of the RNA input amount, ATP-mix dilution, chip lot number and RNA extraction method being used. In contrast, the calls were not robust to changes in labeling method. Overall, the results support that labeling method (figure 14 and 15) and possibly also RNA extraction method, due to large variation in PCA (figure 10 and 11), must be held constant in order to provide for consistent results. Since both of these variables differed between assay I and assay II, changing each of them, or both, in general may prevent a miRNA list obtained in one assay from being able to predict NSCLC samples profiled using another assay, as we found in this study.

## Conclusions

In this study, we demonstrate that some analytic variables are important for the variation in assay performance while others are not. Thus, careful optimization that address all analytical steps and variables can facilitate the introduction of microRNA arrays for prediction of relapse in stage I non-small cell lung cancer (NSCLC). In result, stratification of patients with stage I disease can be improved by prediction of relapse after surgery, potentially allowing to direct intensive surveillance and/or adjuvant therapy toward patients at high risk of relapse (figure 16).

## Methods

### Patients and Tissue Specimens

Patient tumor samples were collected retrospectively from Roswell Park Cancer Institute (RPCI), Buffalo, NY and from Aarhus and Odense University Hospitals in Denmark. The use of all included samples in this study was approved by the institutional review board at Roswell Park Cancer Institute, and, in Denmark, by *Den Videnskabsetiske Komité*. The study was conducted in accordance with the Helsinki declaration. In total 75 NSCLC specimens were included in the study. These comprised 68 stage I NSCLC from a US cohort collected and treated at RPCI [6] and seven NSCLC specimens collected in Denmark. Clinical data were obtained only for the US cohort, from the tumor registry at RPCI and through chart reviews [6]. Approximately half of the patients from the US cohort were known to have had a recurrence. The recurrence-free cases were followed for at least 32 months, with approximately half of them followed for at least 5 years [6]. For the US specimens and for two Danish specimens, tissue cores were sampled from FFPE tissue blocks from areas with > 70% tumor cell content (as verified by HE-stain), and subsequently cores were re-embedded in paraffin. For the remaining four FFPE specimens, tissues sections (20 μm thick) were obtained for the comparison of the two RNA extraction kits. One NSCLC specimen was collected within two hours of surgery and was preserved in *RNAlater *(Ambion, Inc 2130 Woodward St. Austin, TX) with approval by *Den Videnskabsetiske Komité *in Denmark and with informed consent obtained from the patient.

### RNA extraction

In a PubMed http://www.ncbi.nlm.nih.gov/pubmed search using the search terms "miRNA" AND "Cancer" AND "FFPE", 33 publications were retrieved covering the period from 2009 to 2011. Of these, 16 publications described the use of global miRNA profiling, and in over half of these (i.e. in 9 studies), RNA was extracted using the *RecoverAll *kit (Ambion). In the present study we compared two different RNA extraction methods. In addition to the widely used *RecoverAll *kit ("*RA Kit*", Ambion), we included the *High Pure miRNA Isolation Kit *("*HP Kit*", Roche Applied Science, 68298 Mannheim, Germany). For the *HP Kit*, RNA was extracted from deparaffinized and proteinase K-treated FFPE core tissues (20-40 mg) or sections according to the manufacturer's instructions. In approximately one-third of the cases, RNA preparations were of poor quality. Consequently, RNA was extracted again from FFPE tissue. For the *RA Kit*, RNA was extracted from deparaffinized and protease-treated FFPE core tissues (20-40 mg) or sections with on column DNAse digestion according to the manufacturer's instructions. RNA concentration and quality was assessed by absorbance spectrometry and electrophoresis using the NanoDrop (Thermo Fisher Scientific, Wilmington, DE) and Bioanalyzer 2100 (Agilent Technologies) instruments.

### Labeling of RNA

Two different RNA labeling methods were compared in this study; the *FlashTag™ Biotin RNA Labeling Kit *(Genisphere, PA), and the new *HSR *version of the same kit were used according to the manufacturer's instructions unless otherwise stated. *Poly (A)-tailing: *100-800 ng RNA including the small RNA species was used as starting material for the polyA-tailing reaction with different ATP-mix dilutions (from 1:10-1:500). Poly (A)-tailing was performed at 37°C for 15 minutes (GeneAmp PCR System 9700, Applied Biosystems, Foster City, CA). *Ligation: *Using the entire reaction product from the poly (A) tailing reaction, a biotin-labeled 3DNA^®^ dendrimer was ligated to the poly (A) tails using a T4 DNA Ligase and incubated at 25°C for 30 minutes (GeneAmp PCR System 9700, Applied Biosystems^®^) following the manufacturer's instructions.

### Hybridization

Of the 16 studies from the PubMed http://www.ncbi.nlm.nih.gov/pubmed search describing global miRNA profiling, 12 studies used six different commercially available platforms and the remaining four studies used different custom platforms. Between 365 and 847 human miRNAs were profiled using from 20 ng to 5 ug totalRNA as input. In the present study we used the platform with the broadest coverage, i.e. the Afymetrix GeneChip^®^ miRNA Array that interrogates 847 human miRNAs and about an equal number of small non-coding RNAs. Hybridization washing and staining were performed using the Affymetrix GeneChip Hybridization, Wash and Stain Kit (Affymetrix, CA). Briefly, the hybridization cocktail containing the biotin labeled RNA was heated to 99°C for 5 minutes and then to 45°C for 5 minutes (GeneAmp PCR System 9700, Applied Biosystems^®^) before loading onto the Affymetrix probe array cartridge (GeneChip^®^ miRNA Array). The volume of the hybridization cocktail loaded on the chip was changed from 100 ml to 80 ml in order to improve movement/flow of the cocktail in the hybridization chamber, ensuring a better and more even hybridization process. Thus, in effect only 80% of the labeled RNA was placed on the chip. The probe array was incubated for 17 hours at 48°C with constant rotation (60 r.p.m.). The probe array was incubated for 17 h at 48°C at constant rotation (60 r.p.m.). The biotin labeled RNA was stained with a streptavidin-phycoerythrin conjugate and the signals amplified using a biotinylated goat antibody against streptavidin. Finally, the samples were stained with a streptavidin-phycoerythrin conjugate.

### Scanning

The probe arrays were scanned using a confocal laser-scanning microscope (Affymetrix GCS3000Dx2). The readings from the quantitative scanning were analyzed using the Affymetrix Molecular Diagnostics Software (AMDS). The microarray data was deposited in the Array Express public database http://www.ebi.ac.uk/arrayexpress/ and has been assigned accession number E-MTAB-618 (under experiment name: *Laboratory assays for prediction of relapse in stage I non-small cell lung cancer (NSCLC)*).

### RNA input amount

For comparisons, RNA was extracted from a sample collected under conditions where RNA degradation is expected to be minimal. Thus, a sample of a T2 NSCLC post-resection surgical specimen was collected and preserved in *RNAlater *(Ambion) within 2 hours of after surgery. RNA was subsequently extracted using the *HP Kit*. The effect of varying RNA input amounts (80, 100, 160, 400 and 640 ng) for hybridization was examined. In addition, RNA was prepared from ten stage I NSCLC surgical FFPE specimens using the *HP Kit *(100 and 600 ng) and these samples were labeled and hybridized to miRNA arrays. Signal intensities were examined on the chip using laser scanning microscopy. Background intensity ranged from 31 to 36 and was unrelated to the amount of input RNA (results not shown). Subtraction of background intensities did not affect the results (data not shown).

### ATP-mix dilution

The effect of different ATP-mix dilutions (1:10; 1:50 and 1:100) was examined using 100 ng RNA extracted from a single NSCLC specimen using the *HP Kit *and labeled using the *FlashTag™ Biotin RNA Labeling Kit *(Genisphere). In addition, different ATP-mix dilutions (1:50; 1:150 and 1:500) were examined using two preparations of 600 ng RNA extracted from two different NSCLC FFPE specimens using the *RA Kit *and labeled using the *FlashTag™ Biotin HSR RNA Labeling Kit *(Genisphere).

### Different chip lot numbers

Correlations in signal intensities were examined across two different lots of arrays that were hybridized (in triplicates) to 100 ng of labeled RNA (*FlashTag™ Biotin RNA Labeling Kit, *ATP-mix dilution 1:50) from of a single RNA preparation of a T2 NSCLC tumor using the *HP Kit*.

### Comparisons of two different RNA extraction kits

The *High Pure miRNA Isolation Kit *("*HP Kit *", Roche) and *RecoverAll *("*RA Kit", *Ambion) extraction methods were compared in an RNA extraction experiment with four FFPE NSCLC specimens. From each specimen, 20 sections (20 mm thick) were cut, every other slide being used for one extraction method and the remaining slides for the other. For each extraction method, 1000 ng RNA of each specimen was polyadenylated (ATP-mix dilution 1:50) and labeled with biotin using the *FlashTag™ Biotin HSR RNA Labeling Kit *(Genisphere, PA).

### Comparisons of two different labeling kits

The *FlashTag™ Biotin RNA Labeling Kit *(Genisphere), and the new *HSR *version of this kit were compared in an RNA labeling experiment using RNA from four NSCLC specimens from the RPCI cohort extracted using the *HP Kit*. For each labeling kit, 600 ng RNA from each specimen was polyadenylated (ATP-mix dilution 1:50) and labeled with biotin.

### Assay I and II for prognostication in stage I NSCLC

For assay I, total RNA including small RNA was extracted from 68 stage I NSCLC specimens using the *High Pure miRNA Isolation Kit *(Roche) and 600 ng was labeled using the *FlashTag™ Biotin RNA Labeling Kit *(Genisphere). For assay II, total RNA including small RNA was extracted from 63 of the 68 stage I NSCLC specimens using *RecoverAll *(Ambion) and 600 ng was labeled using the *FlashTag™ Biotin HSR RNA Labeling Kit *(Genisphere).

### Prognostic profile based on non-coding small RNA species

We tested the performance of a SVM classifier on both assays using a Monte Carlo approach. First, two-thirds of a data set was randomly chosen and used as a training set. Second, training was done by setting the number of features (i.e. non-coding RNAs) to 30, chosen according to a highest t statistic, MCRestimate package [21], in a LOOCV-loop. The 30 most frequent features were used for training of the SVM classifier. Third, performance was measured on one-third of the data set left out. Finally, the above procedure was repeated 1000 times. A nested LOOCV approach was further used to identify the optimal number of non-coding RNAs in the following way: First, a test sample was held out in the outer loop (leaving N-1 samples). In the inner loop LOOCV on N-1 samples was used to determine the accuracy for a range of selected features. Here, features were first selected based on the highest t statistic mentioned above. Next, selected features were used as input to the SVM classifier to classify each left out sample in turn. Subsequently, the number of features yielding the highest accuracy was used to classify the test sample that was held out in the outer loop. Ultimately, the most frequent number of features yielded the chosen prognostic profile.

### Impact of analytical conditions on the robustness of the prognosticator

To qualify the impact of any variances in the analytical conditions of the assay, the calls (i.e. "recurrence" or "no recurrence") of the prognosticator were examined after varying RNA input amount, ATP-mix dilution, chip lot numbers, RNA extraction kit and RNA labeling kit for eleven selected NSCLC samples. The prognosticator was trained using either 68 (assay I) or 63 (assay II) stage I NSCLC samples using a SVM classifier and the identified prognostic profiles of each assay. Second, the trained SVM classifier was used to predict the outcome and thus examine the robustness of the prognosticator.

### Statistical analysis and Bioinformatics analysis

For testing the effects of RNA amount; ATPmix dilution; extraction kit and labeling kit on signal intensity, background intensity, the numbers of detected probes and probe sets, arrays were pre-processed using Affymetrix miRNA QC Tool 1.0.33 (with *workflow *set to *"*default"). T-tests and ANOVA, assuming equal variances were performed using the R software package [22]. For correlation analysis, data normalization was performed using the justRMA procedure in Bioconductor [21] generating expression indexes (log with base 2) for all human features on the Affymetrix GeneChip miRNA Arrays. Principal component analysis (PCA) was performed using all human non-coding RNAs and the extracted signature for prognostication. For the prognostic profiles we used the raw miRNA data without background correction. We used perfect match probes only and summarized with average difference.

## Competing interests

J Dahlgaard, W. Mazin, T Jensen, A Hansen and S. Knudsen are employees at Medical Prognosis Institute, Denmark, a company that focuses on developing microarray-based technology for cancer treatment. The other authors disclosed no potential competing interest.

## Authors' contributions

JD: Design of study, acquisition of miRNA expression data, statistical data analysis and data interpretation, drafting of the manuscript and final approval. WM: Design of study, statistical data analysis including prognostic models and interpretation, revising the manuscript and final approval. TJ: Design of study; acquisition and interpretation of miRNA expression data; revising the manuscript and final approval. MP: Collection and acquisition of data from FFPE specimens, revising the manuscript and final approval. WB: Collection and acquisition of data from FFPE specimens, revising the manuscript and final approval. AH: Acquisition of miRNA expression data, revising the manuscript and final approval. EK: Acquisition of data, revising the manuscript and final approval. SJHD: Acquisition of data from FFPE specimens, revising the manuscript and final approval. OH: Acquisition of data, revising the manuscript and final approval. HH: Collection and acquisition of data from FFPE specimens, revising the manuscript and final approval. HJD: Acquisition of data; revising the manuscript and final approval. SY Design of study; acquisition and interpretation of clinical data revising the manuscript and final approval. SK Design of study, interpretation and analysis of data including prognostic models, revising the manuscript and final approval.
